# Isolation and identification of phenolic compounds from *Gynura divaricata* leaves

**DOI:** 10.4103/0973-1296.80666

**Published:** 2011

**Authors:** Chunpeng Wan, Yanying Yu, Shouran Zhou, Shuge Tian, Shuwen Cao

**Affiliations:** 1 *State Key Laboratory of Food Science and Technology, Nanchang University, Nanchang-330047, Jiangxi, China*; 2 *Department of Chemistry, Nanchang University, Nanchang - 330 031, Jiangxi, China*; 3 *Jiangxi University of Traditional Chinese Medicine, Nanchang - 330 006, Jiangxi, China*; 4 *Xinjiang Key Laboratory of Famous Prescription and Science of Formulas, Urumqi - 830 011, Xinjiang, China*

**Keywords:** *Gynura divaricata* DC., HPLC-DAD-ESI-MS, phenolic constituents

## Abstract

**Background:**

Phenolic constituents were the principle bioactivity compounds exist in *Gynura divaricata*, little phenolic compounds were reported from the plant previously.

**Materials and Methods:**

60% ethanol extract from the leaves of *Gynura divaricata* were isolated and purified by column chromatography of Silica gel, ODS and Sephadex LH-20, the structures of the isolated compounds were identified by UV, 1H-NMR, 13C-NMR and MS spectroscopic techniques. Additionally, a high-performance liquid chromatography-diode array detector-electrospray ionization-mass (HPLC-DAD-ESI-MS) analytical method was developed to identify some minor constituents in the n-butanol fraction of the ethanol extract of *Gynura divaricata*.

**Results:**

Six flavonols and one Dicaffeoylquinic acid were isolated from the leaves of *Gynura divaricata*, and these compounds were identified as follows: quercetin (1), kaempferol (2), kaempferol-3-O-β-D-glucopyranoside (3), quercetin-3-O-rutinoside (4), kaempferol-3,7-di-O-β-D-glucopyranoside (5), kaempferol-3-O-rutinoside-7-O-β-D-glucopyranoside (6), and 3,5-dicaffeoylquinic acid (7). A total of 13 compounds, including 9 flavonol glycosides and 4 phenolic acids, were tentatively identified by comparing their retention time (RT), UV, and MS spectrum values with those that had been identified and the published data.

**Conclusion:**

This was the first time to use the HPLC-DAD-ESI-MS method to identify the phytochemicals of the genera *Gynura*. Moreover, compounds (6) and (7) have been isolated for the first time from the genus *Gynura*.

## INTRODUCTION

*Gynura* genus belongs to the family Asteraceae, consisting of 12 species in China.[1] Many species are edible medicinal plants and the leaves are also used as a vegetable by the locals in Southwestern China.[2] *G. divaricata* is a traditional Chinese medicinal plant, which is called “Bai Bei San Qi” in Chinese. It has a long history of use for treatment of diabetes in the folk medicine. The ethanol extract of aerial parts of *G. divaricata* was reported to demonstrate hypoglycemic activity in vivo, the flavonoid compounds were the active constituents.[3,4] It also has been reported that many constituents with antiproliferation activity exist in *G. divaricata*.[5,6] The chemical constituents of *G. divaricata* include flavonols, phenolic acids, cerebrosides, polysaccharides, alkaloids, terpenoids, and sterols.[5–10] Flavonols were the principal constituents of the plant, 4 flavonol compounds, including quercetin, isoquercitrin, rutin, and kaempferol-3-O-rutinoside, have been isolated and identified from the aerial parts of the plant.[9] This article herein describes the isolation and structure elucidation of the flavonol and phenolic acid compounds from the ethanol extract of *G. divaricata* DC. leaves by NMR and high-performance liquid chromatography-diode array detector-electrospray ionization-mass spectrometry (HPLC-DAD-ESI-MS) methods.

## MATERIALS AND METHODS

### General

The^1^H-NMR and^13^C-NMR spectra were measured with a Bruker Avance-600 FT-NMR spectrometer (Bruker, Coventry, Germany), with TMS internal standard. HPLC-DAD-ESI-MS were recorded on Waters 2995 Series LC and ZQ-4000 Mass spectrometer (Waters Corporation, Milford, MA, USA). Column chromatography was carried out with Silica gel (Qingdao Marine Chemistry Co. Ltd., 200-300 mesh, Qingdao, China), Sephadex LH-20, and Reverse phase octadecylsilyl (RP-ODS) (Pharmacia Co. Ltd., Minnesota, USA). Thin layer chromatography (TLC) was carried out with Silica gel GF_254_ (Qingdao Marine Chemistry Co. Ltd., Qingdao, China), and the compounds were prepared either by spraying with 10% sulfuric acid ethanol or under UV lamp at 254 nm. HPLC-grade acetonitrile was purchased from Merck Company (Merck, Darmstadt, Germany), other solvents were analytical grade from Sinopharm Chemical Reagent Co. Ltd. (Shanghai, China).

### Plant material

The *Gynura divaricata* plant was obtained in 2009 from Guangdong province, China. A voucher specimen (201001) was deposited at the Department of Chemistry, Nanchang University. The leaves of *G. divaricata* were dried at 40°C in an air oven and finely powdered.

### Extraction and isolation

**Figure 1:**
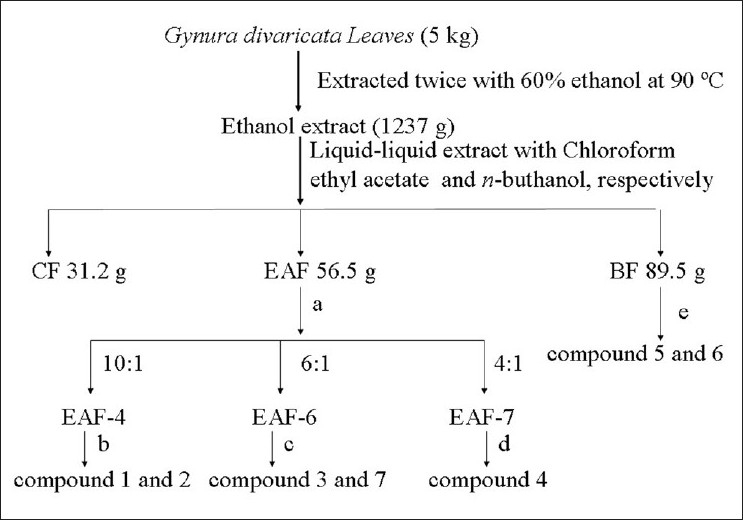
The procedure of extraction and isolation phenolic compounds from *G. divaricata* extracts. (a) Silica gel chromatograph eluted with a mixture of chloroform and methanol (from 100:0 to 4:1); (b) Sephadex LH-20 chromatograph eluted with a mixture of chloroform and methanol (1:1); (c) Sephadex LH-20 column eluted with methanol coupled with RP-ODS column gradient eluted with methanol-water (from 40% to 60%, v/v); (d) Sephadex LH-20 column eluted with methanol, (e) RP-ODS column gradient eluted with methanol-water (from 10% to 50%, v/v) coupled with RP-ODS column and isocratic eluted with methanol-water (18%, v/v)

The weighed portion of the crude drug 5 kg was extracted twice with 60% ethanol (v/v) under reflux at 90°C. The extract was evaporated to dryness *in vacuo*. Extract yield with respect to the dried herb was 25%. The dry extract was suspended in water and subjected to sequential liquid-liquid extraction with chloroform, ethyl acetate (EA), and *n*-butanol, the yield of those 3 extracts were 31.2, 56.5, and 89.5 g, respectively. The EA fraction was chromatographed using flash column on a Silica gel eluted with chloroform-methanol step-gradient (starting with 100:0 to 4:1), eluted fractions were combined on their TLC pattern to yield 8 fractions. The chloroform-methanol fraction (10:1) was chromatographed on a Sephadex LH-20 column eluted with chloroform-methanol (1:1) to yield compounds 1 and 2. The chloroform-methanol fraction (6:1) chromatographed on a Sephadex LH-20 column eluted with methanol and further chromatographed on an RP-ODS column gradient eluted with methanol-water (40%-60%, v/v) gave compounds 3 and 7. The chloroform-methanol fraction (4:1) chromatographed on a Sephadex LH-20 column eluted with methanol yields compound 4 [Figure 1].

The *n*-butanol fraction was chromatographed using flash RP-ODS column gradient eluted with methanol-water (10%-50%, v/v), and the eluted fractions were combined on their HPLC pattern to yield 4 fractions. The methanol-water fraction (25%, v/v) was further chromatographed using flash RP-ODS column and isocratic eluted with methanol-water (18%, v/v) gave compounds 5 and 6. The other minor constituents of *n*-butanol extracts were separated and identified by HPLC-DAD-ESI-MS method.

### HPLC-MS instrument and conditions

The HPLC-DAD-ESI-MS system consists of a Waters 2995 Series LC and ZQ-4000 Mass spectrometer (Waters, USA), equipped with a vacuum degasser, a quaternary pump, an autosampler, a thermostatted column compartment, a diode array detector (DAD), and an ion-trap mass spectrometer with electrospray ionization interface, controlled by Waters 2995 Series LC/ZQ-4000 Trap Software. Shimadzu shimpack VP-ODS (150 mm × 4.6 mm i.d., 5 μm particle size) was used for separation. Solvents for the mobile phase were water-0.1% acetic acid (A) and acetonitrile (B). The gradient elution was 0-30 min, linear gradient 10%-30% B; 30-40 min, linear gradient 30%-100% B. The flow rate was 0.8 mL/min and the column was operated at 30°C. Peaks were detected with the DAD at 254 nm. The ESI negative and positive ionization (NI and PI) total ion current (TIC) modes were used for MS detection. The *m/z* values of the monitored ions were from 100 to 800. The other parameters were as follows: capillary voltage, 3.5 kV; cone voltage, 30 V; extraction voltage, 5 V; RF voltage, 0.5 V; source temperature, 90°C; nitrogen gas flow for desolvation, 300 L/h; and temperature of the nitrogen gas for desolvation, 350°C. Samples for assay were dissolved in 45% MeOH as 3 mg/mL solutions and centrifuged at 12,000 rpm (Beckman, USA) for 15 min to remove particles before injection.

## RESULTS AND DISCUSSION

The compounds were identified using UV, ESI-MS, and NMR spectral data, and determined as quercetin,[11,12] kaempferol,[13,14] kaempferol-3-O-*β*-D-glucopyranoside,[15] quercetin-3-O-rutinoside,[15] kaempferol-3-O-rutinoside-7-O-*β*-D-glucopyranoside,[16,17] kaempferol-3,7-di-O-*β*-D-glucopyranoside,[16] and 3,5-Dicaffeoylquinic acid.[18]

Compound 1 was obtained as a yellow powder, the ESI-MS yielded a quasi-molecular ion peak [M-H]^-^ at *m/z* 301 and [M+H]^+^ at *m/z* 303. The UV spectrum showed λ_max_ at 256 and 370 nm. The^1^H-NMR spectrum showed 2 peaks at δ 6.18 (1H, d, *J* = 2.0 Hz) and 6.40 ppm (1H, d, *J* = 2.0 Hz) consistent with the meta protons H-6 and H-8 on A-ring and an ABX system at 7.68 (1H, d, *J* = 2.2 Hz, H-2’), 7.54 (1H, dd, *J* = 2.0 Hz, 8.4 Hz, H-6’), and 6.88 (1H, d, *J* = 8.4 Hz, H-5’) corresponding to the catechol protons on B-ring. The^13^C-NMR spectrum indicated the presence of 15 carbon atoms, the signal at δ 177.9 was attributed to a carbonyl carbon placed at C-4, and the other signals were compatible with those literatures[11,12] on quercetin.

Compound 2 was obtained as a yellow powder, the ESI-MS yielded a quasi-molecular ion peak [M-H]^-^ at *m/z* 285 and [M+H]^+^ at *m/z* 287. The UV spectrum showed λ_max_ at 265 and 366 nm. The^1^H-NMR spectrum showed 2 peaks at δ 6.17 (1H, d, *J* = 1.8 Hz) and 6.42 ppm (1H, d, *J* = 1.8 Hz) consistent with the meta protons H-6 and H-8 on A-ring and an AA’BB’ system at 8.04 (2H, d, *J* = 8.9 Hz, H-2’, 6’) and 6.93 (2H, d, *J* = 8.9 Hz, H-3’, 5’) corresponding to the protons on B-ring. The MS and^1^ H-NMR data were compatible with the literatures[13,14] of kaempferol.

Compound 3 was obtained as a faint yellow powder, the ESI-MS yielded a quasi-molecular ion peak [M-H]^-^ at *m/z* 447 and [M+H]^+^ at *m/z* 449. The UV spectrum showed λ_max_ at 265 and 346 nm. The^1^ H-NMR spectrum showed 2 peaks at δ 6.21 (1H, d, *J* =1.8 Hz) and 6.44 ppm (1H, d, *J* =1.8 Hz) consistent with the meta protons H-6 and H-8 on A-ring and an AA’BB’ system at 8.04 (2H, d, *J* =8.9 Hz, H-2’, 6’) and 6.89 (2H, d, *J* =8.9 Hz, H-3’, 5’) corresponding to the protons on B-ring. Compound 3 presented the same aglycone signal patterns of compound 2, but the signal at 5.47 (1H, d, *J* =7.2 Hz) followed by other characteristic additional signals indicates the presence of a sugar moiety in compound 3. The hexose was determined to be a glucopyranosyl unit bound to the C-3 position of the aglycone by comparison of proton and carbon upfield shift values with the literature data.[15] Therefore, compound 3 was identified as kaempferol-3-O-*β*-D-glucopyranoside.

Compound 4 was obtained as a faint yellow powder, the ESI-MS yielded a quasi-molecular ion peak [M-H]^-^ at *m/z* 609 and [M+H]^+^ at *m/z* 611. The UV spectrum showed λ_max_ at 258 and 356 nm. The^1^H-NMR spectrum showed 2 peaks at δ 6.20 (1H, d, *J* = 2.0 Hz) and 6.40 ppm (1H, d, *J* = 2.0 Hz) consistent with the meta protons H-6 and H-8 on A-ring and an ABX system at 7.54 (1H, d, *J* = 2.2 Hz, H-2’), 7.59 (1H, dd, *J* = 2.0 Hz, 9.0 Hz, H-6’) and 6.85 (1H, d, *J* = 9.0 Hz, H-5’) corresponding to the catechol protons on B-ring. Compound 4 presented the same aglycone signal patterns of compound 1, two anomeric proton signals at 5.32 (1H, d, *J* =7.2 Hz) and 4.39 (1H, d, *J* = 1.6 Hz) were assignable to H-1 of a *β*-glucosyl proton and to the H-1 of an *α*-rhamnosyl proton, respectively. A methyl signal 0.99 (3H, d, *J* =6.2 Hz) in the high-field region was assigned to rhamnose. In the^13^C-NMR of compound 4, the C-6 signal (68.5) of glucose showed a downfield shift of 7.3 ppm in comparison with the corresponding C-6 signal (61.2) of quercetin-3-O-*β*-D-glucopyranoside,[15] indicating a 1-6 linkage between the glucose and the rhamnose. Therefore, compound 4 was identified as rutin.

Compound 5 was obtained as a faint yellow powder, the molecular formula C_27_H_30_O_16_ was suggested by a mass spectrum with a quasi-molecular ion peak [M-H]^-^ at *m/z* 609, further confirmed by the positive mode mass spectral ions: 611 [M+H]^+^, 449 [M+H-162]^+^, 287 [M+H-162-162]^+^. The UV spectrum showed λ_max_ at 264 and 347 nm typical of a kaempferol glycoside derivative.[15,16,19] In the aromatic region of the^1^ H-NMR spectrum an AA’BB’ system, appearing as two doublets at δ 8.06 (2H, d, *J* = 8.9 Hz, H-2’, 6’) and 6.90 (2H, d, *J* = 8.9 Hz, H-3’, 5’), and two meta coupled doublet protons at δ 6.78 and 6.44 were evident. In the saccharide region of the spectrum two anomeric proton signals were present as large doublets at δ 5.48 and 5.08. The coupling constant (*J* = 7.2 Hz) of the two anomeric protons characteristic for *β*-configuration. The downfield shift of the H-6 and H-8 proton, as well as downfield shift of the corresponding carbons at δ 99.8 and δ 94.9, with respect to the corresponding signals of aglycone, suggested the linkage with the sugar moiety across the oxygen of the C(7)-OH group.[17] The chemical shift (δ 5.48) suggested that the other sugar moiety is directly attached to the C(3)-OH group, further confirmed by the upfield shift of the signal assigned to C-3 (133.9).[15,16] Acid hydrolysis of compound 5 afforded kaempferol and glucose comparison with the authentic samples on TLC. From the above data, compound 5 was identified as kaempferol-3,7-di-O-*β*-D-glucopyranoside.

Compound 6 was obtained as a faint yellow powder, the molecular formula C_33_H_40_O_20_ was suggested by a mass spectrum with a quasi-molecular ion peak [M-H]^-^ at *m/z* 755. The UV and^1^ H-NMR spectrum of compound 6 was similar to that of 5, suggesting that compound 6 also was a kaempferol glycoside derivative, the only difference being the presence of a methyl signal (δ 0.99) in the high-field region, which was assigned to rhamnose, further confirmed by the doublet proton at δ 4.44, was assigned to the anomeric proton of rhamnose with a coupling constant (*J* = 1.6 Hz) characteristic for *α*-linked rhamnose. The^13^C-NMR spectrum of 6 confirms that compound 6 is a triglycoside of kaempferol [Table 1]. Careful examination of the^13^C-NMR spectrum of 6 showed that the signal assigned to the glucose C-6 [Table 1] was shifted downfield by appropriately 6 ppm (from 61.3 to 67.3) confirming that the rhamnose moiety linkage to the glucose C-6.[17] From the above data, compound 6 was identified as kaempferol-3-O-rutinoside-7-O-*β*-D-glucopyranoside.

**Table 1:** The 1H-NMR and 13C-NMR spectrum data of kaempferol-3,7-di-O-*β*-d-glucopyranoside and kaempferol-3-O-rutinoside-7-O-*β*-d-glucopyranoside (DMSO-d6)

Atom	Kaempferol-3,7-di-O-*β*-d-glucoside			Kaempferol-3-O-rutinoside-7-O-*β*-d-glucoside		
	^1^H		^13^C	^1^H		^13^C
	δ(ppm)	J(Hz)	δ(ppm)	δ(ppm)	J(Hz)	δ(ppm)
2			156.5			156.5
3			133.9			140.0
4			178.1			178.1
4a			105.9			161.4
5			161.3			106.1
6	6.44 d	2.0	99.8	6.45 d	2.0	99.8
7			163.3			163.4
8	6.78 d	2.0	94.9	6.76 d	2.0	95.1
8a			157.3			157.8
1’			121.1			121.2
2’	8.06 d	8.6	131.4	8.01 d	8.8	131.5
3’	6.90 d	8.6	115.6	6.90 d	8.8	115.6
4’			160.6			160.6
5’			115.6			115.6
6’			131.4			131.5
3-O-Rutinoside						
G1	5.48 d	7.2	101.2	5.35 d	7.2	101.7
G2			74.6			74.7
G3			76.9			76.9
G4			70.4			70.4
G5			78			77.7
G6			61.3			67.3
R1				4.44 d	1.6	101.2
R2						70.8
R3						71.1
R4						72.3
R5						68.7
R6				0.99 d	6.2	18.2
7-O-Glucoside						
G’1	5.08 d	7.2	100.2	5.08 d	7.2	100.3
G’2			73.5			73.6
G’3			76.9			76.3
G’4			70.0			70.1
G’5			77.6			76.9
G’6			61.1			61.1

Compound 7 was obtained as amorphous powder, the ESI-MS yielded a quasi-molecular ion peak [M-H]^-^ at *m/z* 515 and [M+H]^+^ at *m/z* 517. The UV spectrum showed λ_max_ 327, 294 (sh), and 248 nm (sh), which were characteristic of caffeic acid derivatives. In the^1^ H-NMR spectrum, two caffeoyl groups were presented at δ 7.50 (1H, d, *J*=16.0 Hz, H-7’), 7.43 (1H, d, *J*=16.0 Hz, H-7”), 7.05 (2H, brs, H-2’, 2”), 7.01 (2H, brd, *J*=2.0 Hz, H-6, 6”), 6.78 (1H, d, *J*=8.0 Hz, H-5’), 6.76 (1H, d, *J*=8.0 Hz, H-5”), 6.26 (1H, dd, *J*=16.0 Hz, H-8’), 6.14 (1H, dd, *J*=16.0 Hz, H-8”). A quinic acid moiety was presented at 5.42 (1H, brs, H-3), 5.18 (1H, m, H-5), 3.86 (1H, brs, H-4), 2.20 (2H, m, H-6), 2.01(2H, m, H-2). The^1^H-NMR data were in agreement with the literature[18] and compound 7 was identified as 3,5-Dicaffeoylquinic acid.

**Figure 2:**
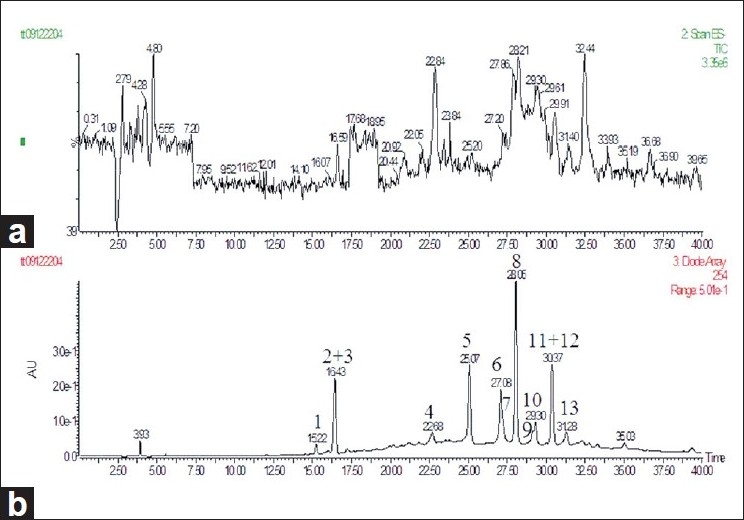
The TIC chromatogram of negative model (a) and HPLC-DAD chromatogram of the n-butanol fraction of *G. divaricata* extracts (b)

**Figure 3:**
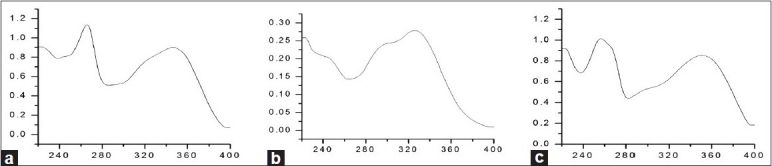
The typical UV spectrum of Kaempferol glucopyranoside derivative (a), Dicaffeoylquinic acid (b), and Quercetin glucopyranoside derivative (c)

An HPLC-DAD-ESI-MS method was developed to identify the minor phytochemical constituents of *n*-butanol fraction of *G. divaricata* extract. The chromatogram of MS TIC in negative mode is shown in Figure 2a. As shown in Figure 2b, 13 major peaks were detected under the HPLC conditions with DAD detection at 254 nm. Peaks of 2, 3, and 11, 12 were co-eluted in the present conditions and unequivocally determined to be kaempferol-3,7-di-O-*β*-D-glucopyranoside, kaempferol-3-O-rutinoside-7-O-D-glucopyranoside, 3,5-Dicaffeoylquinic acid, and kaempferol-3-O-*β*-D-glucopyranoside, respectively. And peak 5 was identified as quercetin-3-O-rutinoside. All of those 5 peaks were identified by comparing the retention time (RT), UV [Figure 3], and ESI-MS values with isolation compounds. The other compounds were tentatively identified based on the UV adsorption value, *m/z* value, and elution order compared with the published data.

Peak 1 was believed to be an unidentified minor flavonol glycoside due to its low concentration in the extract, peak 1 and 3 are a pair of isomers, the UV (λ_max_) and *m/z* values [Table 2] were similar to peak 3 (identified as kaempferol-3-O-rutinoside-7-O-*β*-D-glucopyranoside). The elution order of peak 1 being prior to peak 3 [Table 2] suggested that rutinose of peak 3 was substituted by a robinobiose, and the structure of peak 1 was proposed to be kaempferol-3-O-robinobioside-7-O-*β*-D-glucopyranoside.[20]

**Table 2:** HPLC-DAD-ESI-MS (positive and negative ionization TIC modes) fingerprint of *n*-butanol fraction of *G. divaricata* extracts

Peak No.	*t*_R_ (min)	*λ*_max_(nm)	Product ions (ESI-, *m/z*)	Product ions (ESI+, *m/z*)	Identification of compounds
1	15.22	265, 346	755 [M-H]^-^		Kaempferol-3-O-robinobioside-7-O-*β*-D-glucoside
2	16.43	264, 347	609 [M-H]^-^	611 [M+H]^+^ 449 [M+H-162]^+^ 287 [M+H-162-162]^+^	Kaempferol-3,7-di-O-*β*-D-glucoside
3	16.51	264, 347	755 [M-H]^-^	757 [M+H]^+^ 611 [M+H-146]^+^ 449 [M+H-146-162]^+^ 287 [M+H-146-162-162]^+^	Kaempferol-3-O-rutinoside-7-O-*β*-D-glucoside
4	22.68	247, 307	337 [M-H]^-^ 191 [M-H-146]^-^	339 [M+H]^+^ 147 [M+H-192]^+^	*p*-Coumaoylquinic acid
5	25.07	254, 356	609 [M-H]^-^	611 [M+H]^+^ 465 [M+H-146]^+^ 303 [M+H-146-162]^+^	Quercetin-3-O-rutinoside
6	27.08	256, 354	463 [M-H]^-^	465 [M+H]^+^ 303 [M+H-162]^+^	Quercetin-3-O-*β*-D-glucoside
7	27.26	265, 346	593 [M-H]^-^	595 [M+H]^+^ 449 [M+H-146]^+^ 287 [M+H-146-162]^+^	Kaempferol-3-O-robinobioside
8	28.05	265, 347	593 [M-H]^-^	595 [M+H]^+^ 449 [M+H-146]^+^ 287 [M+H-146-162]^+^	Kaempferol-3-O-rutinoside
9	29.12	265, 346	447 [M-H]^-^	449 [M+H]^+^ 287 [M+H-162]^+^	Kaempferol-3-O-*β*-D-galacoside
10	29.30	248, 327	515 [M-H]^-^ 353 [M-H-162].	499 [M+H-18]^+^ 163 [M+H-162-192]^+^	3,4-Dicaffeoylquinic acid
11	30.37	248, 325	515 [M-H]^-^ 353 [M-H-162].	499 [M+H-18]^+^ 163 [M+H-162-192]^+^	3,5-Dicaffeoylquinic acid
12	30.37	265, 347	447 [M-H]^-^	449 [M+H]^+^ 287 [M+H-162]^+^	Kaempferol-3-O-*β*-D-glucoside
13	31.28	248, 325	515 [M-H]^-^ 353 [M-H-162].	499 [M+H-18]^+^ 163 [M+H-162-192]^+^	4,5-Dicaffeoylquinic acid

HPLC-DAD-ESI-MS: High-performance liquid chromatography-diode array detector-electrospray ionization-mass spectrometry, TIC: Total ion current, Identification was supported by comparison with reference standards where available and by correlation with previous literature reports. Peaks 2, 3 and 11, 12 were co-eluted. Peak numbers and retention times (TR) refer to HPLC chromatograms in Figure 2b

Peak 4 yielded a [M-H]^-^ ion at *m/z* 337, and [M+H]^+^ ion at *m/z* 339, [M+H-192]^+^ ion at *m/z* 147. The UV spectrum showed λ_max_> at 307, 293 (sh), and 247 nm (sh), which is characteristic of a Cinnamic acid derivative[19,21]; hence, the structure of peak 4 was proposed to be p-coumaroylquinic acid.[19,21]

Peak 6 yielded a [M-H]^-^ ion at *m/z* 463, and [M+H]^+^ ion at *m/z* 465, [M+H-162]^+^ ion at *m/z* 303. The UV spectrum showed λ_max_ at 255 and 356 nm, suggesting that this as a quercetin glycoside.[21] By examining the known flavonol glycoside in the genus *Gynura*, isoquercitrin was consistent with the above data. And the elution order of isoquercitrin was in agreement with the compound prior toKaempferol-3-O-robinobioside (peak 7) and afterward with rutin (peak 5).[22–24] Thus, peak 6 was tentatively identified as isoquercitrin.

Peak 7 and 8 were a pair of isomers. Both of them gave a [M-H]^-^ ion at *m/z* 593, and [M+H]^+^ ion at *m/z* 595, [M+H-146]^+^ ion at *m/z* 449, [M+H-146-162]^+^ ion at *m/z* 287. The UV spectrum showed λ_max_ at 265 and 347 nm, which suggested peak 7 and 8 were kaempferol glycoside derivatives.[15–17,19] By examining the known kaempferol glycoside in the genus *Gynura*, Kaempferol-3-O-robinobioside and kaempferol-3-O-rutinoside were consistent with the above data.[25] The elution order in HPLC of Kaempferol-3-O-robinobioside being prior to kaempferol-3-O-rutinoside has been reported by many in the literature.[26,27] Thus, peak 7 and 8 were identified as Kaempferol-3-O-robinobioside and kaempferol-3-O-rutinoside, respectively.

Peak 9 yielded a [M-H]^-^ ion at *m/z* 447, and [M+H]^+^ ion at *m/z* 449, [M+H-162]^+^ ion at *m/z* 287. The UV spectrum showed λ_max_ at 265 and 346 nm, suggesting this as a kaempferol glycoside. So peak 9 is an isomer of kaempferol-3-O-*β*-D-glucopyranoside (peak 12). Thus, peak 9 was tentatively identified as kaempferol-3-O-*β*-D-galacopyranoside.

Peak 10, 11, and 13 are isomers. Both of them gave a [M-H]^-^ ion at *m/z* 515, [M-H-162]^-^ ion at *m/z* 353, and [M+H]^+^ ion at *m/z* 517, [M+H-18]^+^ ion at *m/z* 499, [M+H-162-192]^+^ ion at *m/z* 163. The 3 compounds also had similar UV absorptions with maxima at 327, 294 (sh), and 248 nm (sh), which is characteristic of caffeic acid derivatives.[28–31] Peak 11 was isolated by the chromatography column and identified as 3,5-Dicaffeoylquinic acid by the NMR and ESI-MS spectrum data. According to the elution order in HPLC of Dicaffeoylquinic acid reported in the literature,[31–33] 3,4-Dicaffeoylquinic acid is prior to 3,5-Dicaffeoylquinic acid, which is prior to 4,5-Dicaffeoylquinic acid, in a sequence. Thus, peak 10 and 13 were tentatively identified as 3,4-Dicaffeoylquinic acid and 4,5-Dicaffeoylquinic acid, respectively.

The flavonoid and phenolic acid compounds were affected by the concentration of extraction ethanol. The single-factor experiment showed that 60% ethanol was suitable to extract the phenolic constituents from the plant. The levels of phenolic contents were decreased as the concentration of ethanol increased. Chloroform was used to remove the nonpolar constituents, while little extracts were obtained using diethyl ether and petroleum ether. The ethyl acetate extracts showed powerful antioxidant activity and highest total phenolic content. HPLC analysis showed that ethyl acetate extracts only shared 3 principal peaks, and the kaempferol-3-O-*β*-D-glucopyranoside was the major constituent. However, *n*-butanol extract shared numerous flavonoid compounds, while the total phenolic was lower. In order to fully elaborate the phenolic compounds of the extract from *G. divaricata*, the extracts of ethyl acetate and *n*-butanol were isolated using chromatograph column and HPLC-DAD-ESI-MS method. To our best knowledge, the present study is the first report of the isolation and identification of triglycoside of kaempferol and Dicaffeoylquinic acid from the leaves of *G. divaricata*. And we also developed a HPLC-DAD-ESI-MS method to separate and identify the minor constituents of the *n*-butanol extracts. The bioactive evaluation of the isolated compounds and the crude drug deserved further research.

## CONCLUSION

Seven phenolic compounds were isolated and identified from the leaves of *G. divaricata*, and the structures were fully elucidated by the spectrum methods. HPLC-DAD-ESI-MS method was used to identify the other 8 minor phenolic constituents of the *n*-butanol extracts. This was the first time to use the HPLC-DAD-ESI-MS method to identify the phytochemicals of the genera *Gynura*, and kaempferol-3-O-rutinoside-7-O-*β*-D-glucopyranoside and 3,5-Dicaffeoylquinic acid were identified for the first time from the genus *Gynura*.
